# Innate immunity in chemotherapy-induced peripheral neuropathy: recent advances

**DOI:** 10.3389/fpain.2025.1642306

**Published:** 2025-10-28

**Authors:** Nana Dong, Tongtong Lin

**Affiliations:** ^1^ Department of Oncology, Yantaishan Hospital, Yantai, Shandong, China; ^2^ State Key Laboratory of Technologies for Chinese Medicine Pharmaceutical Process Control and Intelligent Manufacture, Nanjing University of Chinese Medicine, Nanjing, Jiangsu, China

**Keywords:** chemotherapy-induced peripheral neuropathy, innate immune system, neurotoxicity, quality life, macrophage

## Abstract

Chemotherapy-induced peripheral neuropathy (CIPN) is a common dose-limiting side effect in patients undergoing chemotherapy. Many commonly used chemotherapeutic agents simultaneously induce neurotoxicity and modulate the immune system. Emerging evidence highlights a critical role of the innate immune system in the development of various neuropathic pain conditions. As a natural immune defense mechanism formed during phylogenetic evolution, innate immunity elicits a robust response during CIPN pathogenesis. This review summarizes the roles of the innate immune system—including the skin barrier, innate immune cells, and innate immune molecules—in the context of CIPN.

## 1 Introduction

Advances in cancer therapeutics have markedly improved patient survival rates; however, chemotherapy-induced peripheral neuropathy (CIPN) remains a prevalent, dose-limiting toxicity that significantly compromises quality of life and therapeutic continuity. Clinical epidemiological data indicate that the incidence of CIPN ranges between 50% and 90% (1–5). A comprehensive meta-analysis of 77 studies conducted across 28 countries, encompassing 10,962 patients, estimated a pooled prevalence of chronic painful CIPN at 41.22%, with substantial heterogeneity observed among studies. Subgroup analyses revealed higher prevalence in patients receiving platinum compounds or taxanes, and in those diagnosed with lung cancer (6).

Despite its high burden, current evidence-based preventive or disease-modifying interventions are lacking. According to the latest clinical practice guidelines issued by the American Society of Clinical Oncology (ASCO), duloxetine is the only pharmacological agent recommended for the management of painful CIPN (7). However, randomized controlled trials have failed to demonstrate prophylactic efficacy. A recent prevention study in colorectal cancer patients showed that duloxetine (30 mg or 60 mg daily) did not reduce the incidence of acute oxaliplatin-induced peripheral neuropathy vs. placebo, with no significant difference in patient-reported symptoms. Moreover, the duloxetine arm exhibited poorer adherence, with fatigue and nausea as common adverse events (8). Consequently, duloxetine is currently endorsed solely for symptomatic pain relief and not for CIPN prevention.

Multiple pharmacological approaches have been investigated—including anticonvulsants (e.g., gabapentin), tricyclic antidepressants (e.g., amitriptyline), vitamin B supplementation, calcium–magnesium infusions, and diverse chemoprotective agents—yet none have demonstrated definitive neuroprotective effects in clinical trials (7). As a result, most guideline-based strategies for CIPN management are extrapolated from treatments for other neuropathic pain conditions, such as opioids, antiepileptics, and serotonin–norepinephrine reuptake inhibitors (SNRIs) (9). However, their efficacy in alleviating CIPN-specific symptoms remains limited. In current clinical practice, modification of chemotherapy dosage, or even discontinuation, remains the primary recourse for severe or progressive disease cases.

Traditionally, pathogenesis research has primarily focused on the direct neurotoxic effects of chemotherapeutic agents, including mitochondrial dysfunction, ion channel abnormalities, and oxidative stress pathways (10). However, recent years have witnessed a critical paradigm shift in research approaches, with investigators actively pursuing novel interventions that can effectively preserve peripheral nerve function without compromising the antitumor activity of chemotherapeutic drugs. Preserving the integrity of neural function not only significantly enhances patients' quality of life but may also optimize overall oncological treatment outcomes by improving therapeutic tolerance. This research transition has positioned non-neuronal components within the nervous system—particularly neuroimmune interaction mechanisms—as pivotal breakthrough points for elucidating CIPN pathogenesis and developing targeted therapies (11–13).

In the complex pathogenic network of CIPN, aberrant activation of the innate immune system has been demonstrated to play a central regulatory role. As an evolutionarily conserved defense mechanism, innate immunity precisely identifies chemotherapy-induced damage-associated molecular patterns (DAMPs) through pattern recognition receptors (PRRs), thereby triggering cascading immune responses. Clinical studies have demonstrated significantly elevated levels of innate immune-derived pro-inflammatory mediators such as TNF-α and IL-1β in the peripheral blood of CIPN patients. Animal models further reveal that various innate immune cells, including macrophages, neutrophils, and mast cells, undergo specific recruitment to injured nerve tissues following chemotherapeutic exposure. Upon PRR-mediated recognition of DAMPs in the microenvironment, these immunocytes become activated and subsequently release abundant pro-inflammatory mediators (e.g., cytokines, chemokines, and reactive oxygen species), establishing a persistent neuroinflammatory milieu (4). Of particular significance, this immune-neural crosstalk not only induces neuroinflammatory states but also contributes to peripheral sensitization through modulation of nociceptor electrophysiological properties (14). The prolonged persistence of such pathological alterations may represent the mechanistic basis underlying the intractable chronic pain associated with CIPN.

To advance understanding of these mechanisms and their therapeutic implications, this review aims to comprehensively elucidate the contributions of the innate immune system to CIPN development, focusing on its multifaceted components as potential therapeutic targets. Specifically, we seek to: (1) delineate the skin's role as a dynamic neuro-immunological interface, where barrier disruption and keratinocyte-mediated DAMP signaling initiate local inflammatory cascades; (2) examine the recruitment and activation of key innate immune cells, including macrophages, neutrophils, mast cells, and microglia, which orchestrate neuroinflammatory milieus through phagocytosis, mediator release, and crosstalk with neural elements; (3) analyze the impact of cytokines (e.g., TNF-α, IL-1β, IL-6) and chemokines (e.g., CX3CL1/CX3CR1, CCL2/CCR2) in sustaining immune cell trafficking, nociceptor modulation, and chronic pain states; and (4) integrate these insights to highlight translational opportunities, such as targeted inhibition of PRR pathways or mediator antagonists, to restore neuro-immune homeostasis without compromising anticancer efficacy.

## 2 Materials and methods

### 2.1 Search strategy

We performed a comprehensive narrative literature search to identify preclinical and clinical studies reporting pathophysiological mechanisms and therapeutic strategies for chemotherapy-induced peripheral neuropathy (CIPN), focusing on innate immune system involvement. Searches were conducted in PubMed, Web of Science, and Embase up to September 2025.

### 2.2 Inclusion criteria

Study type—Preclinical (*in vivo* rodent models) or clinical studies. Chemotherapeutic agents—Paclitaxel, oxaliplatin, cisplatin, vincristine.

Focus—Mechanistic investigations into innate immune cell involvement (macrophages, mast cells, microglia, neutrophils), chemokine signaling, skin barrier alterations, and interventions for CIPN.

Outcome—Includes histological evidence, molecular signaling pathways, neurobehavioral changes, or therapeutic interventions.

### 2.3 Presentation of results

Data were systematically extracted into a structured table recording: (1) Authors, year, reference number; (2) Study design and model; (3) Chemotherapy agent(s); (4) Immune cell type(s) or molecular pathways involved; (5) Intervention and outcomes; (6) Findings were organized by immune cell category (macrophages, mast cells, microglia, neutrophils) and innate immune mediators, with separate sections for skin barrier involvement and clinical evidence; (7) Key mechanistic and therapeutic findings are presented in summary tables and integrated into the discussion.

## 3 The role of skin

The skin, as the first line of defense between the body and external environment, has evolved beyond a simple physical barrier to become a dynamic neuro-immunological regulatory hub. Contemporary research demonstrates that this organ integrates mechanical protection, microbial defense, and neurosensory functions through a sophisticated cellular network comprising both immune and non-immune cells, serving as a central mediator in maintaining tissue homeostasis and responding to external insults (15, 16). This multifaceted protective system relies not only on physical barrier structures and antimicrobial molecules but also involves intricate immune surveillance and neural signaling mechanisms.

In the pathogenesis of chemotherapy-induced peripheral neuropathy (CIPN), skin-mediated neuro-immune interactions have recently emerged as critical contributors (17). Recent studies have revealed three pathological mechanisms by which the skin contributes to CIPN: Current evidence identifies three key pathological mechanisms through which the skin exacerbates CIPN: First, chemotherapeutic agents compromise stratum corneum integrity, triggering intraepidermal nerve fiber (IENF) degeneration and sensory nerve terminal exposure a finding corroborated by clinical biopsy studies (18–20). Damaged keratinocytes recognize damage-associated molecular patterns (DAMPs) via Toll-like receptor 4 (TLR4) signaling, subsequently upregulating pro-inflammatory cytokines [e.g., interleukin-1β (IL-1β) and tumor necrosis factor-α (TNF-α)] (21–23), while concurrently dysregulating antimicrobial peptide secretion (including β-defensins), thereby amplifying localized neuroinflammation (24–26). Second, experimental models reveal that paclitaxel and vincristine significantly enhance epidermal infiltration of PGP9.5-positive Langerhans cells, which directly promote pain sensitization through nitric oxide and pro-inflammatory mediator release (20). Third, imbalances in cutaneous neurotrophic factors—particularly reduced nerve growth factor (NGF) expression alongside elevated neurotrophin-3 (NT-3) levels—disrupt nerve repair mechanisms. Clinical evidence suggests that the high-concentration capsaicin exerts a biphasic therapeutic effect in CIPN. In the initial phase, high-concentration capsaicin induces a functional “defunctionalization” of nociceptive C fibers by disorganizing their cytoskeletal structure, leading to transient degeneration of intraepidermal nerve endings and temporary reduction in pain signaling. This early deactivation step is followed by a regenerative phase, in which a secondary increase in intraepidermal and subepidermal nerve fiber density reflects re-growth and functional recovery of the cutaneous nerve network. Mechanistically, capsaicin patch treatment has been shown to restore the disrupted NGF/NT-3 ratio to physiological levels, thereby supporting nerve repair processes. Post-treatment skin biopsies consistently demonstrate significant increases in both intraepidermal and subepidermal nerve fiber density (27, 28), indicating that targeted cutaneous interventions can not only promote structural regeneration but also effectively rebalance neuro-immune homeostasis. These findings underscore the therapeutic potential of skin barrier-protective strategies—including reinforcement of stratum corneum integrity and modulation of local immune responses—as innovative avenues for CIPN management. The key pathological mechanisms of skin involvement in CIPN and corresponding therapeutic strategies are systematically summarized in Table 1.

**Table 1:** Summary of skin-related studies in chemotherapy-induced peripheral neuropathy.

First author (Year, Ref)	Study type	Model/Subjects	Chemotherapy agent	Main findings	Intervention
Boyette-Davis et al. (18)	Preclinical	Rat skin	Paclitaxel	Paclitaxel damaged SC integrity, triggered IENF loss; minocycline prevented IENF loss and reduced hyperalgesia	Minocycline
Ko et al. (19)	Preclinical	Rat skin	Paclitaxel	Loss of peptidergic IENF associated with neuropathic pain behaviors	Peptidergic IENFs
Siau et al. (20)	Preclinical	Rat skin	Paclitaxel, Vincristine	Chemotherapy increases PGP9.5-positive langerhans cell infiltration, and the activated Langerhans cells release NO and pro-inflammatory mediators that lead to pain sensitization	—
Anand et al. (27); Anand & Bley (28)	Clinical	CIPN patients	—	Loss of skin nerve fibers with reduced NGF and elevated NT-3 impairs repair, while 8% capsaicin restores the NGF/NT-3 balance and nerve fiber density.	Capsaicin 8% patch

## 4 Innate immune cells

### 4.1 Macrophages

Macrophages originate from myeloid progenitor cells in the bone marrow and constitute essential components of the mononuclear phagocyte system. As principal effector cells of innate immunity, these versatile immune cells execute three cardinal functions: (i) pathogen and cellular debris clearance through phagocytosis; (ii) antigen processing and presentation to initiate adaptive immune responses; and (iii) cytokine secretion to modulate inflammatory processes (29).

Emerging evidence demonstrates that macrophages within the peripheral nervous system, particularly those localized in the dorsal root ganglion (DRG), exert pivotal regulatory roles in neuropathic pain pathogenesis (30, 31). Experimental investigations reveal that following peripheral nerve injury, the ipsilateral DRG demonstrates a marked increase in macrophage infiltration, primarily from circulating monocyte-derived populations. Yu et al. demonstrated that axotomy-induced neuropathic pain in both male and female murine models promotes DRG macrophage expansion, which is indispensable for neuropathic pain initiation and maintenance (32). Notably, this dynamic macrophage response within the DRG microenvironment also represents a key pathogenic mechanism in CIPN.

Current research establishes that paclitaxel-induced CIPN correlates with significant macrophage infiltration in the DRG. Longitudinal immunofluorescence analyses demonstrate that CD68+ macrophage accumulation in L4-L5 DRG segments begins to increase three days post-chemotherapy, reaches a zenith at day 14, and sustains elevated levels through day 21 (33). These activated macrophages exhibit characteristic morphological transformations, including cytoplasmic vacuolation, mitochondrial swelling, and increased cell volume, while preferentially localizing around neuronal somata (34). Importantly, this DRG macrophage infiltration pattern extends beyond paclitaxel, having been documented in CIPN models induced by platinum-based agents (cisplatin, oxaliplatin) and vinca alkaloids (vincristine) (35–38).

The pathogenic contribution of macrophages to CIPN involves complex molecular regulatory networks. Toll-like receptor (TLR) signaling pathways represent critical mediators in this process. Paclitaxel administration triggers robust TLR4 signaling activation, which in turn propagates the p38/NF-*κ*B inflammatory cascade in a TLR4-dependent manner, directly contributing to pain sensitization. In oxaliplatin-induced CIPN models, neutrophil extracellular traps (NETs) within the DRG microenvironment engage TLR7 and TLR9 receptors on macrophages to activate the NLRP3 inflammasome, culminating in IL-18 release and nociceptor hyperexcitability (39). Intriguingly, TLR9 signaling exhibits sexually dimorphic characteristics in paclitaxel-induced CIPN: it demonstrates pronounced disease-promoting effects in male mice while showing attenuated regulatory activity in females (40).

Beyond canonical TLR pathways, macrophages orchestrate CIPN pathogenesis through multifaceted molecular mechanisms. High-mobility group box 1 (HMGB1), a prototypical damage-associated molecular pattern (DAMP) molecule, demonstrates dual-pathogenic effects in CIPN: it accelerates disease progression via ROS/p38 MAPK/NF-κB/HAT signaling activation, while simultaneously forming the HMGB1-TLR4-PI3K/Akt-MMP-9 signaling axis to synergistically amplify neuroinflammatory responses (38). Pro-inflammatory cytokines such as TNF-α and IL-1β secreted by activated macrophages directly induce sensory neuron damage and pain sensitization through RIP3/MLKL-mediated necroptotic pathways (41). Additionally, recent studies have shown that Schwann cell-derived galectin-3 modulates macrophage infiltration patterns, contributing to CIPN development (42).

Based on these mechanistic insights, multiple experiments have confirmed that targeted interventions—such as blocking TLR4 signaling with lipopolysaccharide (LPS) antagonists, inhibiting macrophage recruitment with MCP-1 neutralizing antibodies, or depleting macrophages using macrophage-depleting agents—can effectively suppress abnormal macrophage activation and pathological infiltration, thereby significantly alleviating chemotherapy-induced mechanical allodynia (33, 43, 44). These findings provide a crucial theoretical foundation for developing novel therapeutic strategies for CIPN.

### 4.2 Neutrophils

In recent years, the mechanisms by which neutrophils contribute to chemotherapy-induced peripheral neuropathy have gradually garnered attention. Chemotherapeutic agents such as oxaliplatin can induce intestinal barrier damage, leading to the leakage of LPS and HMGB1 into the bloodstream, which subsequently activates neutrophils infiltrating the DRG to form NETs (45). These NETs exacerbate nerve damage through a dual mechanism: on the one hand, the tissue factor (TF) captured by NETs triggers microthrombus formation, resulting in microcirculatory disturbances and a hypoxic microenvironment in the DRG and peripheral nerves. This process aggravates axonal injury and mechanical hypersensitivity via the HIF-1α/MMP-9 signaling axis (46); on the other hand, NETs activate the TLR7/TLR9 receptors on macrophages, triggering NLRP3 inflammasome activation and promoting IL-18 release. This, in turn, induces abnormal phosphorylation of the GluN2B subunit at the Tyr1472 site in spinal dorsal horn neurons, ultimately leading to central sensitization. Clinical data further reveal elevated plasma levels of NETs markers (such as citrullinated histone H3) in CIPN patients, which positively correlate with pain intensity (39).

Targeting this mechanism, enzyme-based therapies for NETs degradation represent another promising intervention strategy. Wang et al. innovatively developed an ischemia-homing peptide (SHp, CLEVSRKNC)-modified DNase1 nanodrug (Cy5-SHp-DNase1) that specifically accumulates in hypoxic areas with NETs deposition, enabling highly efficient targeted degradation. Compared to conventional DNase1, this engineered enzyme exhibits dual functions: serving as an *in vivo* hypoxia probe to visualize NETs deposition sites while precisely degrading NETs to improve microcirculatory dysfunction. In animal studies, SHp-DNase1 significantly alleviated oxaliplatin-induced mechanical allodynia, demonstrating superior efficacy to either standard DNase1 or the anticoagulant hirudin. This patented formulation shows strong clinical translation potential and may overcome the limited effectiveness of traditional anticoagulants against NETs-associated thrombosis (46).

### 4.3 Mast cells

Mast cells are tissue-resident immune cells widely distributed in the skin, around blood vessels of visceral organs, and throughout the peripheral nervous system. Located at the interface between the body and external environment, they serve as rapid responders to environmental threats (47). Although these cells are best known for their roles in allergic reactions and anti-parasitic immunity, recent studies have revealed their crucial function in neuro-immune interactions. Mast cells can sense tissue damage and inflammatory signals, bidirectionally modulating neural activity and immune responses through the release of mediators such as histamine, proteases, and cytokines (48, 49). Benefiting from their close anatomical and functional connections with neurons, mast cells have emerged as important bridges linking the nervous and immune systems.

In the pathogenesis of CIPN, neuroinflammation has been confirmed as a critical factor in disease development. As a key regulator of neuroinflammation, mast cells participate in the pathological process of CIPN through multiple pathways: On one hand, chemotherapeutic agents can directly activate perineural mast cells, triggering their degranulation and releasing large amounts of inflammatory mediators. These mediators can not only directly alter the excitability of sensory neurons but also recruit immune cells such as macrophages and neutrophils to form an inflammatory cascade (14); On the other hand, neurotrophic factors (e.g., NGF) secreted by mast cells play a dual role in neuroprotection and nerve injury, and disruption of this dynamic balance ultimately leads to neurological dysfunction and hyperalgesia (50).

Emerging research has further elucidated the molecular mechanisms of mast cells in chemotherapy-induced neuropathic pain. In oxaliplatin-treated models, significant mast cell degranulation and increased mast cell numbers were observed in the plantar epidermis of mice. Genetic studies revealed that congenital mast cell-deficient mice showed remarkable resistance to oxaliplatin-induced mechanical allodynia (51). Similarly, vincristine was found to trigger mast cell degranulation, particularly promoting histamine release, which subsequently induced thermal hyperalgesia and cold allodynia through the H1/H2 receptor pathway (52). Paclitaxel, through a unique “histamine-H1 receptor-PKC-TRPV1” signaling axis, enhanced thermal sensitivity of TRPV1 channels in dorsal root ganglion neurons (53).

Beyond the histamine system, studies have also uncovered the mechanisms of novel mast cell-derived algogenic mediators. Oxaliplatin was found to significantly elevate skin serine protease activity, an effect markedly attenuated in mast cell-deficient mice. Crucially, the analgesic effects of serine protease inhibitors confirmed the pivotal role of these mediators in mechanical allodynia (51). Notably, the mast cell-specific protease tryptase can induce hyperalgesia by activating protease-activated receptor-2 (PAR-2) receptors through a neurokinin-1 receptor-dependent pathway, while simultaneously promoting neuronal release of pain-related neuropeptides such as g calcitonin gene-related peptide (CGRP) and substance P (SP) (54, 55). In paclitaxel-treated models, peripheral tissue tryptase activity showed significant positive correlation with thermal hyperalgesia severity. Both PAR-2 antagonists and inhibitors targeting its downstream signaling pathways (including PLCβ, PKCε and PKA) demonstrated promising analgesic effects (56).

The sphingolipid metabolite sphingosine-1-phosphate (S1P) is another critical mediator in mast cell-mediated pain regulation (57, 58). Its synthesis is tightly controlled by Fc*ε*RI signaling, and it acts in an autocrine manner via S1P1/S1P2 receptors to modulate mast cell degranulation, chemotaxis, and neuropathic pain (59). Preclinical studies show that S1P1 receptor blockade alleviates cancer-induced bone pain and chemotherapy-induced neuropathy. Notably, the S1P1 modulator fingolimod exhibits neuroprotective and anti-inflammatory effects in both paclitaxel and oxaliplatin models, offering a promising therapeutic strategy for CIPN (60).

### 4.4 Microglia

Studies have demonstrated that microglial activation is a key pathological feature in oxaliplatin-induced neuropathic pain, making it a novel potential therapeutic target for alleviating chemotherapy-related neuropathic pain (61–63). In animal models of oxaliplatin-induced CIPN, significant activation of spinal microglia has been observed. Notably, intrathecal administration of the microglial inhibitor minocycline effectively ameliorates oxaliplatin-induced CIPN symptoms. Temporal analysis reveals that microglial activation peaks on day 7 post-treatment but gradually returns to baseline levels by days 14 and 21 (64). Further research indicates that oxaliplatin treatment induces microglial activation in the mouse spinal cord, accompanied by upregulated expression of various inflammatory mediators, including pro-inflammatory cytokines (IL-1β, IL-6, and TNF-α), inflammation-related enzymes (COX-2 and iNOS), and signaling molecules (p-ERK and p-NF-κB), a phenomenon validated in the BV-2 microglial cell line (65).

In cisplatin-induced neuropathic pain models, studies have uncovered a more complex regulatory network. Cisplatin activates the spinal TREM2/DAP12 signaling pathway, triggering microglial inflammatory responses and significantly elevating the expression of inflammatory markers such as IL-1β, IL-6, TNF-α, iNOS, and CD68. This persistent neuroinflammation can lead to intraepidermal nerve fiber (IENF) loss and may induce structural and functional changes in DRG neurons, ultimately mediating the development of pain symptoms. Importantly, blocking microglial activation or inhibiting TREM2 signaling effectively prevents IENF loss and significantly alleviates cisplatin-induced mechanical allodynia (66). Additionally, the P2Y12-SFK-p38 signaling pathway promotes spinal central sensitization through an IL-18-dependent mechanism, providing a theoretical basis for developing analgesic strategies targeting the P2Y12 receptor (67). Recent studies have also revealed that abnormal cholesterol accumulation in microglia is a critical mechanism underlying neuropathic pain in cisplatin-induced CIPN models. Targeted modulation of cholesterol metabolism pathways not only restores the normal state of inflammatory lipid rafts but also reprograms microglial phenotypes, thereby producing long-lasting analgesic effects (68). In vincristine-induced CIPN mouse models, intrathecal administration of the γ-secretase inhibitor DAPT alleviates neuropathic pain symptoms by inhibiting the NOTCH signaling pathway, which subsequently downregulates the activity of the microglial CX3CR1/p38/MAPK pathway (69). The key pathological mechanisms of innate immune cells involvement in CIPN and corresponding therapeutic strategies are systematically summarized in Table 2.

**Table 2:** Summary of innate immune cells studies in chemotherapy-induced peripheral neuropathy.

First author (Year, Ref)	Study type	Model/Subjects	Chemotherapy agent	Main findings	Intervention
Macrophages studies in hemotherapy-induced peripheral neuropathy					
Zhang et al. (33)	Preclinical	Rat DRG	Paclitaxel	TLR4–MCP-1–macrophage axis in the DRG drives inflammation, sensory fiber loss, and neuropathy	Macrophage-depleting agents
Peters et al. (34)	Preclinical	Rat DRG	Paclitaxel	Neuronal and glial injury is accompanied by macrophage and microglial activation, driving neuropathic pain behaviors	—
Kiguchi et al. (35)	Preclinical	Mouse sciatic nerve/DRG	Vincristine	Peripheral macrophage-derived IL-6 essential for mechanical allodynia	IL-6 neutralizing antibody
Zhang et al. (36)	Preclinical	Mouse DRG	Cisplatin	HDAC6 inhibition reverses hypersensitivity via IL-10 production and macrophage-mediated effects	HDAC6 inhibitor
Yang et al. (37)	Preclinical	Mouse sciatic nerve	Oxaliplatin	HSP70-TLR4-p38-TF-HIF-1α pathway in macrophages and sciatic nerves contributes to ischemic/hypoxic injury	Hirudin
Gu et al. (38)	Preclinical	Mouse DRG	Oxaliplatin	HMGB1-TLR4-PI3K/Akt-MMP-9 axis mediates macrophage–neuron crosstalk in the DRG, promoting neuropathogenesis	NAC
Lin et al. (39)	Preclinical	Mouse DRG	Oxaliplatin	neutrophil extracellular traps activate NLRP3 inflammasomes in macrophages, inducing IL-18 release and mechanical hyperalgesia	IL-18BP; EPA
Luo et al. (40)	Preclinical	Mouse DRG	Paclitaxel	Sex-dimorphic TLR9 signaling in macrophages drives mechanical allodynia in males but not in females.	TLR9 antagonist ODN 2088
Ma et al. (41)	Preclinical	Mouse DRG	Paclitaxel	Macrophage infiltration into the DRG promotes TNF-α and IL-1β release, triggering neuronal necroptosis via the RIP3/MLKL pathway	Clodronate disodium
Koyanagi et al. (42)	Preclinical	Mouse sciatic nerve/DRG	Paclitaxel	Schwann cell-derived galectin-3 enhances macrophage infiltration and pain hypersensitivity	Galectin-3 inhibitor TD-139
Zhang et al. (43)	Preclinical	Mouse DRG	Paclitaxel	MCP-1/CCR2 signaling recruits macrophages, causes sensory fiber loss, and blocking this pathway reduces mechanical hypersensitivity	MCP-1 neutralizing antibody
Li et al. (44)	Preclinical	Mouse	Paclitaxel	TLR4 activation triggers MAPK/NF-κB signaling, promoting macrophage recruitment and activation	MAPK inhibition
Neutrophils studies in chemotherapy-induced peripheral neuropathy					
Lin et al. (39)	Preclinical	Mouse DRG	Oxaliplatin	Neutrophils contribute to oxaliplatin-induced peripheral neuropathy by forming extracellular traps that activate macrophage NLRP3 inflammasomes, leading to IL-18–mediated hyperalgesia	EPA
Jia et al. (45)	Preclinical	Mouse DRG	Oxaliplatin	Neutrophils promote chemotherapy-induced peripheral neuropathy by forming and accumulating extracellular traps that contribute to neuroinflammation via the gut-blood-DRG axis	Fucoidan
Wang et al. (46)	Preclinical	Mouse DRG	Oxaliplatin	Engineered Cy5-SHp-DNase1 nanodrug targeted hypoxic NETs deposition; visualized NET sites & degraded NETs; improved microcirculation and reduced mechanical allodynia	SHp-DNase1
Mast cells studies in chemotherapy-induced peripheral neuropathy					
Sakamoto et al. (51)	Preclinical	Mouse plantar skin	Oxaliplatin	Mast cells release serine proteases that activate PAR2 on sensory neurons, a process driven by capsaicin-sensitive primary afferents	Camostat mesilate, FSLLRY-NH2
Jaggi et al. (52)	Preclinical	Mouse	Vincristine	Mast cell degranulation releases histamine, which primarily activates H1 receptors, driving mechanical and heat hyperalgesia as well as cold allodynia	Cromoglycate, promethazine
Gao et al. (53)	Preclinical	Mouse DRG	Paclitaxel	Mast cell-derived histamine contributes to pain, and the mast cell stabilizer quercetin alleviates symptoms by inhibiting histamine release and suppressing PKC*ε* and TRPV1 upregulation in the spinal cord and DRG	Quercetin
Kume et al. (56)	Preclinical	Mouse hind paw skin and DRG	Paclitaxel	Mast cell-derived proteases activate PAR2 on sensory neurons, driving mechanical allodynia and promoting neuroinflammation	PAR2 antagonist C781
Janes et al. (60)	Preclinical	Rat spinal cord	Paclitaxel, Oxaliplatin	Ceramide–S1P/S1PR1 signaling mediates neuroinflammation in the spinal dorsal horn, potentially amplified by mast cell-derived S1P	S1PR1 antagonists (NIBR-14/15)
Microglia studies in chemotherapy-induced peripheral neuropathy					
Di Cesare Mannelli et al. (63)	Preclinical	Mouse spinal cord	Oxaliplatin	Loss of α7 nAChR and increased spinal microglia are observed, while α7 nAChR agonists (R)-ICH3 and PNU-282987 alleviate pain and protect neural integrity	α7 nAChR agonists (R)-ICH3 and PNU-282987
Di Cesare Mannelli et al. (64)	Preclinical	Mouse spinal cord	Oxaliplatin	Synergistic signaling between spinal microglia and astrocytes drives nociceptive sensitization.	Minocycline
Lee et al. (65)	Preclinical	Mouse spinal cord	Oxaliplatin	Spinal microglial activation and inflammatory signaling in the dorsal horn mediate neuropathic symptoms.	Syringaresinol
Hu et al. (66)	Preclinical	Mouse spinal cord	Cisplatin	Persistent activation of spinal microglia via enhanced TREM2/DAP12 signaling drives pain progression	TREM2 neutralizing antibody
Chen et al. (67)	Preclinical	Mouse spinal cord	Cisplatin	Spinal microglia mediate nociceptive processing via P2Y12–SFK-p38 signaling and IL-18-driven central sensitization	P2Y12 inhibitor MRS2395
Navia-Pelaez et al. (68)	Preclinical	Mouse spinal cord	Cisplatin	Cholesterol accumulation in spinal microglia triggers TLR4 inflammaraft-mediated neuroinflammation and neuropathic pain	apoA-I binding protein
Qin et al. (69)	Preclinical	Rat spinal cord	Vincristine	Spinal microglial activation via the Notch signaling pathway contributes to hyperalgesia	γ-secretase inhibitor (DAPT)

## 5 Cytokines

Chemotherapy-induced inflammatory responses are considered potential drivers of nociceptive processes in CIPN. Pro-inflammatory and chemotactic factors released after chemotherapy are recognized as key mechanisms regulating neuro-immune interactions, with downstream cytokine effects serving as critical triggers for neuroinflammation in the sensory nervous system (70–72). Chemotherapy leads to significant cytokine release, including TNF-α, IL-1β, and IL-6. In oxaliplatin-induced rat CIPN models, elevated levels of IL-1β, IL-6, and TNF-α in the periaqueductal gray (PAG) correlate with reduced γ-aminobutyric acid (GABA). Further experiments demonstrate that blocking pro-inflammatory cytokine receptors in the PAG of oxaliplatin-treated rats largely restores GABA concentrations and alleviates mechanical allodynia and cold hyperalgesia (73). Pro-inflammatory cytokines not only cause axonal damage and promote neuron-immune responses, increasing the release of bradykinin, serotonin, and histamine, but also exert direct toxic effects on neural cells by mediating the activity of neurons and glia through specific receptors (74, 75).

In breast cancer patients, serum levels of IL-6 and soluble IL-6 receptor (sIL-6R) are significantly higher in those with CIPN compared to those without symptoms, suggesting that persistent CIPN and reduced quality of life may be linked to increased levels of the inflammatory mediator IL-6 and its soluble receptor (76). Studies have shown that the IL-8 signaling pathway contributes to peripheral pain development in oxaliplatin- and paclitaxel-induced CIPN models, and the IL-8 inhibitor DF2726A effectively alleviates CIPN symptoms (77).

Compared to inflammatory cytokines such as TNF-α, IL-1β, and IL-6, the role of IL-17 in pain remains less understood. Recently, IL-17 was found to regulate inflammatory responses associated with nerve injury-induced neuropathic pain. In neuropathic pain models, IL-17 levels are upregulated in injured nerves, and IL-17 receptors (IL-17R) are detected in most neurons in dorsal root ganglion (DRG) sections and cultured DRG neurons. IL-17-deficient mice exhibit reduced mechanical pain sensitivity compared to wild-type mice after methadone injection or partial sciatic nerve ligation. Emerging research reports that astrocyte-derived IL-17 suppresses inhibitory synaptic transmission in spinal pain circuits and drives chemotherapy-induced neuropathic pain. IL-17 not only enhances excitatory postsynaptic currents (EPSCs) in somatostatin-expressing neurons in mouse spinal cord slices but also inhibits inhibitory postsynaptic currents (IPSCs). Selective knockout of IL-17R in spinal neurons reduces paclitaxel-induced hypersensitivity. In DRGs, the expression of IL-17R in nociceptive neurons is both necessary and sufficient for paclitaxel-induced neuronal hyperexcitability (78).

In mouse models, serum and DRG levels of interleukin-20 (IL-20) fluctuate with paclitaxel treatment. Blocking IL-20 with neutralizing antibodies or genetic deletion of its receptor prevents CIPN, mitigates peripheral nerve damage, and suppresses inflammatory responses, including macrophage infiltration and cytokine release. Mechanistically, targeting IL-20 improves paclitaxel-induced peripheral neuropathy by inhibiting neuroinflammation and restoring calcium homeostasis (79). The key pathological mechanisms of cytokines involvement in CIPN and corresponding therapeutic strategies are systematically summarized in Table 3.

**Table 3:** Summary of cytokines studies in chemotherapy-induced peripheral neuropathy.

First author (Year, Ref)	Study type	Model/Subjects	Chemotherapy agent	Main findings	Intervention
Xu et al. (73)	Preclinical	Rat (PAG region)	Oxaliplatin	Elevated IL-1β, IL-6, and TNF-α and increased membrane expression of their receptors in the periaqueductal gray impair descending inhibitory GABAergic pathways	Cytokine receptor antagonists
Starkweather (76)	Clinical	CIPN patients	Mixed chemotherapy	Women with painful chemotherapy-induced peripheral neuropathy showed elevated IL-6 and soluble IL-6R and reduced soluble gp130	—
Brandolini et al. (77)	Preclinical	Mouse DRG	Oxaliplatin, Paclitaxel	IL-8 signaling contributed to peripheral pain, IL-8 inhibitor reduced CIPN symptoms	IL-8 inhibitor DF2726A
Luo et al. (78)	Preclinical	Mouse spinal cord/DRG	Paclitaxel	Glia-produced IL-17 acts via IL-17R to enhance excitatory and suppress inhibitory synaptic transmission in spinal pain circuits, driving paclitaxel-induced neuropathic pain through neuron-glial interactions and neuronal hyperexcitability	IL-17R knockout/blockade
Chen et al. (79)	Preclinical	Mouse DRG	Paclitaxel	Elevated IL-20 drives pathology, and blocking IL-20 prevents neuropathy by suppressing neuroinflammation and restoring Ca²^+^ homeostasis without compromising anticancer efficacy	IL-20 neutralizing antibody

## 6 Chemokines

Chemokines are regulators of peripheral immune cell trafficking and are also expressed on neurons and glial cells in the central nervous system. As potential mediators and contributors to CIPN pain signaling, chemokines and their receptors—including CX3CL1/CX3CR1, CCL2/CCR2, CXCL1/CXCR2, CXCL12/CXCR4, and CCL3/CCR5—exhibit altered expression under CIPN pathological conditions. Chemokine receptor antagonists have been shown to alleviate neuropathic pain behaviors, suggesting that innate immune activation may represent a broad mechanistic basis for CIPN (80).

Paclitaxel has been found to significantly upregulate the chemokine CX3CL1 in A-fiber sensory neurons, thereby inducing macrophage infiltration into DRG. Intrathecal or systemic administration of CX3CL1-neutralizing antibodies blocks paclitaxel-induced macrophage recruitment, DRG neuronal apoptosis, and abnormal pain. Mechanistically, CX3CL1 inhibition reduces p38 MAPK activation in macrophages, thereby suppressing neuronal apoptosis and mechanical pain development. These findings provide novel evidence that CX3CL1-recruited macrophages contribute to paclitaxel-induced DRG neuronal damage and painful peripheral neuropathy (81). In vincristine-induced CIPN models, upregulated endothelial adhesion promotes infiltration of circulating CX3CR1^+^ monocytes into the sciatic nerve. At the endothelial-nerve interface, CX3CL1 activates CX3CR1+ monocytes to generate reactive oxygen species (ROS), which subsequently activate TRPA1 receptors in sensory neurons, eliciting pain responses. Notably, CX3CR1-deficient mice exhibit delayed pain onset after vincristine treatment. Targeting CX3CR1 with antagonists or inhibiting CX3CL1 proteolytic shedding (*via* ADAM10/17 or cathepsin S) may serve as a therapeutic strategy for chemotherapy-induced peripheral pain (82).

The CCL2 (MCP-1)/CCR2 axis plays a critical role in paclitaxel-induced CIPN. Studies report increased expression of MCP-1 and its receptor CCR2 in DRGs of paclitaxel-treated animals. MCP-1 elevates intracellular calcium in large- and medium-sized DRG neurons, while anti-MCP-1 antibodies or CCR2 antisense oligonucleotides attenuate mechanical allodynia and prevent intraepidermal nerve fiber (IENF) loss. The MCP-1/CCR2 pathway is thus a promising therapeutic target (43). In oxaliplatin-induced CIPN, CCL2/CCR2 expression peaks at day 4 post-treatment and normalizes by day 15. Intrathecal anti-CCL2 antibody therapy prevents mechanical hypersensitivity and reverses established pain, highlighting the translational potential of this pathway (80).

In vincristine models, CX3CR1+ monocytes in the sciatic nerve mediate mechanical allodynia. While Cx3cr1^−/−^ mice eventually develop pain due to compensatory CCL2/CCR2 upregulation, CCR2 antagonists effectively mitigate pain, revealing crosstalk between these chemokine pathways (83).

Paclitaxel increases spinal dorsal horn (SDH) microglia and upregulates CCL3/CCR5. Intrathecal CCL3-neutralizing antibodies prevent and reverse mechanical pain. P2X7 receptors (P2X7Rs) on microglia facilitate CCL3 release, and the P2X7R antagonist A438079 shows both prophylactic and therapeutic efficacy (84).

Vincristine and paclitaxel upregulate CXCL12 in DRGs. This chemokine, released from neuronal central terminals, attracts T cells and monocytes via CXCR4 activation, increasing intracellular calcium and promoting immune cell chemotaxis (85).

Weighted Gene Co-Expression Network Analysis (WGCNA) identified CXCL10, CCL21, CCR2, CXCR4, TLR4, NPY1R, and GALR2 as hub genes linked to CIPN status, severity, and susceptibility. Chemokine regulators (e.g., CXCL10, CCL21, CCR2, CXCR4) are associated with peripheral immune cell trafficking in CIPN (86). The key pathological mechanisms of chemokines involvement in CIPN and corresponding therapeutic strategies are systematically summarized in Table 4.

**Table 4:** Summary of chemokines studies in chemotherapy-induced peripheral neuropathy.

First Author (Year, Ref)	Study type	Model/subjects	Chemotherapy agent	Main findings	Intervention
Huang et al. (81)	Preclinical	Mouse DRG	Paclitaxel	CX3CL1 upregulation recruits macrophages to the dorsal root ganglion, triggers neuronal apoptosis via p38 MAPK activation, and drives mechanical allodynia	CX3CL1-neutralizing antibody
Old et al. (82)	Preclinical	Mouse DRG/spinal cord	Vincristine	CX3CL1 activates CX3CR1^+^ monocytes, which trigger TRPA1-dependent sensory neuron activation; blocking CX3CR1 or CX3CL1 shedding prevents pain development	CX3CR1 antagonist
Illias et al. (80)	Preclinical	Rat DRG	Oxaliplatin	CCL2-CCR2 signaling in the dorsal root ganglion drives pain and can be prevented or reversed by pathway inhibition	Anti-CCL2 antibody
Zhang et al. (43)	Preclinical	Mouse DRG	Paclitaxel	MCP-1/CCR2 signaling recruits macrophages, causes sensory fiber loss, and its blockade reduces mechanical hypersensitivity	MCP-1 neutralizing antibody
Montague et al. (83)	Preclinical	Mouse DRG	Vincristine	In vincristine-induced allodynia in CX3CR1-deficient mice, upregulated CCL2-CCR2 signaling in monocytes arises from CX3CR1-CCR2 interaction and p38 MAPK activation, promoting pronociceptive cytokine release.	CCR2 antagonist RS-102895
Ochi-ishi et al. (84)	Preclinical	Mouse spinal cord	Paclitaxel	spinal microglial P2X7 receptor activation triggers CCL3 release, and blocking CCL3-CCR5 signaling or P2X7 receptors prevents or reverses symptoms	CCL3-neutralizing antibody; P2X7R antagonist A438079
Zhou et al. (86)	Clinical + Bioinformatic	CIPN patients	Cisplatin	WGCNA analysis identified CXCL10, CCL21, CCR2, and CXCR4 as hub genes associated with symptom severity and susceptibility	—

## 7 Outlook and perspectives

Advances in anti-tumor therapies have markedly improved patient survival, yet chemotherapy-induced peripheral neuropathy remains a major complication that compromises cancer patients' quality of life. Beyond causing neuropathic pain, paresthesia, and other debilitating symptoms, CIPN often necessitates chemotherapy dose modification or discontinuation, thereby undermining oncological treatment efficacy. This condition imposes a multidimensional burden—physical, psychological, and social—on affected individuals. However, current preventive and therapeutic strategies for CIPN remain largely inadequate.

Recent studies highlight the central role of aberrant innate immune activation in CIPN pathogenesis. This review has addressed two key dimensions: (1) mechanistic pathways by which innate immunity drives CIPN progression; and (2) the potential clinical utility of therapeutic approaches targeting innate immune components. These mechanistic insights provide a foundation for developing new interventions that preserve anti-tumor potency while alleviating symptom burden. Nonetheless, several critical challenges hinder progress. First, distinct chemotherapeutic agents (e.g., oxaliplatin, cisplatin, vincristine) trigger unique patterns of immune activation and cellular recruitment, complicating the development of broadly applicable therapies. Second, most preclinical studies employ tumor-free animal models, which fail to account for the modulatory effects of the tumor microenvironment on neuroimmune dynamics.

In addition, emerging evidence implicates adaptive immune responses in CIPN recovery. For example, CD8^+^ T cells within the dorsal root ganglion (DRG) are essential for resolution of paclitaxel-induced mechanical allodynia; T cell-deficient mice exhibit prolonged symptom duration compared to wild-type counterparts (87). In cisplatin-induced CIPN models, T cells mediate the protective effects of selective histone deacetylase 6 (HDAC6) inhibitors against mechanical hyperalgesia and mitochondrial dysfunction in DRG neurons (88). Furthermore, paclitaxel-exposed T cells stimulate macrophages to secrete interleukin-10 (IL-10) via interleukin-13 (IL-13), and macrophage-derived IL-10 engages IL-10 receptors (IL-10R) on DRGs to counteract paclitaxel-induced peripheral neuropathy (89). Based on current evidence, future research priorities should include: (1) Developing precision interventions tailored to the distinct immune signatures of specific chemotherapeutic agents; (2) Validating therapeutic targets in tumor-bearing animal models to enhance clinical relevance; (3) Elucidating synergistic mechanisms between innate and adaptive immune systems; (4) Exploring novel therapies targeting key effector cells, such as DRG monocytes/macrophages and spinal microglia. Advancing these research directions will support the design of innovative strategies that effectively control CIPN while safeguarding anti-tumor efficacy—ultimately achieving the dual objectives of improving cancer treatment outcomes and enhancing patient quality of life.
